# Research on an Improved Segmentation Recognition Algorithm of Overlapping *Agaricus bisporus*

**DOI:** 10.3390/s22103946

**Published:** 2022-05-23

**Authors:** Shuzhen Yang, Bowen Ni, Wanhe Du, Tao Yu

**Affiliations:** 1 School of Mechatronic Engineering and Automation, Shanghai University, Shanghai 200444, China; nibowen@shu.edu.cn (B.N.); yutao@shu.edu.cn (T.Y.); 2 School of Intelligent Manufacturing and Control Engineering, Shanghai Polytechnic University, Shanghai 201209, China; whdu@sspu.edu.cn

**Keywords:** overlapping, *Agaricus bisporus*, segmentation recognition algorithm, image edge gradient feature, contour segmentation, grouping recognition

## Abstract

The accurate identification of overlapping *Agaricus bisporus* in a factory environment is one of the challenges faced by automated picking. In order to better segment the complex adhesion between *Agaricus bisporus*, this paper proposes a segmentation recognition algorithm for overlapping *Agaricus bisporus*. This algorithm calculates the global gradient threshold and divides the image according to the image edge gradient feature to obtain the binary image. Then, the binary image is filtered and morphologically processed, and the contour of the overlapping *Agaricus bisporus* area is obtained by edge detection in the Canny operator, the convex hull and concave area are extracted for polygon simplification, and the vertices are extracted using Harris corner detection to determine the segmentation point. After dividing the contour fragments by the dividing point, the branch definition algorithm is used to merge and group all the contours of the same *Agaricus bisporus*. Finally, the least squares ellipse fitting algorithm and the minimum distance circle fitting algorithm are used to reconstruct the outline of *Agaricus bisporus*, and the demand information of *Agaricus bisporus* picking is obtained. The experimental results show that this method can effectively overcome the influence of uneven illumination during image acquisition and be more adaptive to complex planting environments. The recognition rate of *Agaricus bisporus* in overlapping situations is more than 96%, and the average coordinate deviation rate of the algorithm is less than 1.59%.

## 1. Introduction

The factory production of *Agaricus bisporus* is relatively mature, and the daily output of the larger *Agaricus bisporus* plant can reach more than ten tons [1,2]. Because the background of the mushroom bed is complex and diverse, the size and shape of the *Agaricus bisporus* community vary greatly, and there are more complex adhesions between them, so the picking and locating becomes difficult [3]. At present, the picking of *Agaricus bisporus* in factory production mainly depends on manual labor, but manual picking has the problems of large labor, low efficiency, high cost and inconsistent standards [4]. With the increase in mushroom production, mushroom farming is currently facing the problems of labor shortage and rising costs to maintain sustainability [5]. Therefore, it is an inevitable trend to realize the automatic picking of *Agaricus bisporus*. With the development of robotics, robotic harvesting of *Agaricus bisporus* has been researched [6,7,8,9]. Among them, machine vision technology is the key technology in the *Agaricus bisporus* picking robot [10,11].

At present, mushroom recognition and positioning methods based on machine vision are widely used. Yu Gaohong [12] proposed to start from the central coordinate points of each central area, search for mushroom boundary points along the radius of different angles, and store the found mushroom boundary points in the corresponding dynamic linked list to achieve the independent division of each mushroom. Yang Yongqiang [13] proposed to use Harris corners as texture features to filter the background, accurately extract the foreground targets, search for the regional extreme points in the foreground distance map, and then use the marker-based watershed algorithm to achieve the adhesion of the mushroom segmentation, and finally, ellipse fitting achieves positioning. For segmentation recognition of overlapping target objects, UECS [14] uses a morphological segmentation method to obtain the label of each object, which can be used to segment particles in all shapes, but if the degree of overlap is high, the segmentation results may be affected. In [15,16,17,18], through polygon approximation and ellipse fitting, concave point extraction was used to segment overlapping objects. Although this method is effective for regularly shaped objects, objects with shapes that deviate from the ellipse are problematic.

In [19,20], a twice-watershed algorithm is proposed to segment *Agaricus bisporus* during size grading, but for overlapping *Agaricus bisporus*, it seems there are issues in. Sun Jingwei [21] adopted a submergence algorithm on depth images for mushroom segmentation, but how to obtain a high-quality depth image in the case of overlapping is a practical problem. In [22,23,24], object recognition based on a deep learning framework was developed for human activity and intention recognition, and the framework demonstrated superior performance. However, this kind of algorithm has not been widely used in *Agaricus bisporus* recognition, which also provides a new idea.

In this paper, the segmentation identification of overlapping *Agaricus bisporus* is studied. We highlight the contributions of this paper as follows.

(1) We adopt gradient feature to reduce the influence of illumination variance.
(2) Considering grouping the segmented contours as combinatorial optimization, we propose a branch definition algorithm to merge and group the dispersed outlines of the same *Agaricus bisporus*.
(3) To solve arc segmentation with different curvatures and lengths for reconstruction of *Agaricus bisporus* contours, we exploit two algorithms: the least square ellipse fitting algorithm for high curvature or long length and the minimum distance circle fitting for low curvature or short length.

## 2. Segmentation of Overlapping *Agaricus bisporus*

**Figure 1:**
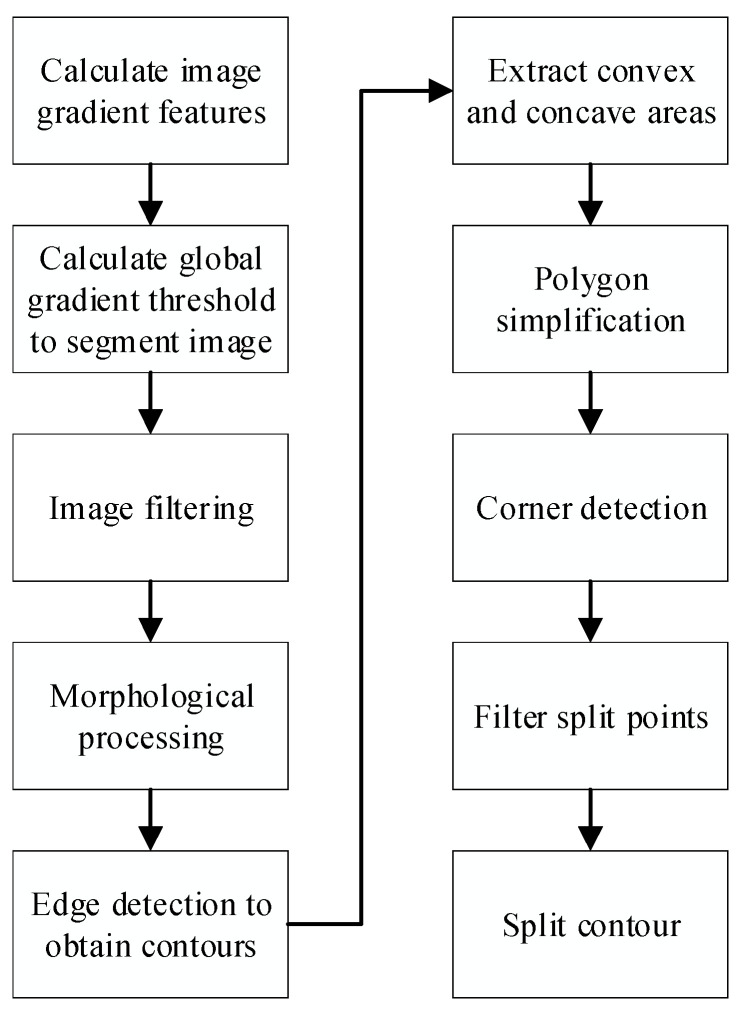
Flow chart of segmentation of *Agaricus bisporus*.

Due to the large difference in soil height and uneven illumination under the industrialized environment of *Agaricus bisporus*, according to the edge gradient characteristics in the image, a global gradient threshold is calculated to segment the image to obtain a binary image. Then, the binary image is filtered and morphologically processed, the contour is obtained by edge detection in the Canny operator, the convex hull of the overlapping *Agaricus bisporus* area is extracted, and the concave area is extracted to simplify the polygon. Finally, Shi-Tomasi corner detection is used to extract the vertices and determine the segmentation points, and the overlapping outlines of *Agaricus bisporus* are segmented. The specific process is shown in Figure 1.

### 2.1. Segmentation Based on Image Edge Gradient

Due to the complicated and diverse planting environment of *Agaricus bisporus*, the difference in soil height and the uneven illumination, the traditional threshold segmentation cannot fully extract the *Agaricus bisporus* area, which affects the accuracy of the edge fitting of *Agaricus bisporus*. Therefore, combining the image gradient feature to calculate the global gradient threshold to segment the image can obtain the bisporus area more completely.

Using the gradient characteristics in the image [25,26], the gradients in the X direction and the Y direction are calculated separately; that is, the first-order differentiation of the image is obtained. Assuming that the image is f(x,y), the gradients of the X direction and Y direction of the f(x,y) at the coordinate point (x,y) are:

(1) $$\frac{\partial f\left(x,y\right)}{\partial x}=f\left(x+1,y\right)-f\left(x,y\right)\;$$

(2) $$\frac{\partial f\left(x,y\right)}{\partial y}=f\left(x,y+1\right)-f\left(x,y\right)\;$$

**Figure 2:**
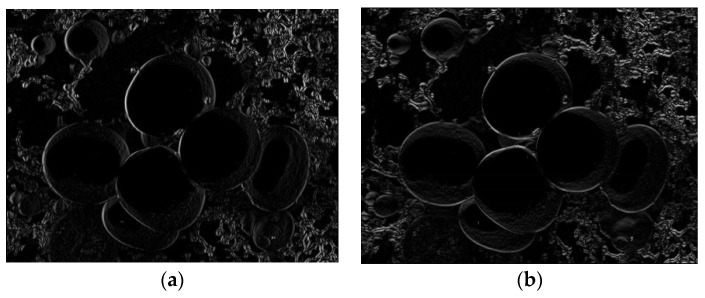
Image edge gradient. (**a**) *X* direction gradient map; (**b**) *Y* direction gradient map.

The gradient images of f(x,y), corresponding to the X and Y directions, are shown in Figure 2.

The maximum value of the gradient M(x,y) is calculated according to the gradient in the X and Y directions:

(3) $$M\left(x,y\right)=Max\left(\frac{\partial f\left(x,y\right)}{\partial x},\frac{\partial f\left(x,y\right)}{\partial y}\right)$$

According to the maximum value, the global gradient threshold T is calculated:

(4) $$T=\frac{\sum_{x=0}^{R_{x}}\sum_{y=0}^{R_{y}}P\left(x,y\right)M\left(x,y\right)}{\sum_{x=0}^{R_{x}}\sum_{y=0}^{R_{y}}M\left(x,y\right)}\;$$

where P(x,y) is the gray value at (x,y), $R_{x},\;R_{y}$ are the number of rows and columns of the image.

The global gradient threshold T is used for image segmentation to extract the *Agaricus bisporus* region. The segmented binary image G(x,y) is:

(5) $$G\left(x,y\right)=\{\begin{array}{cc} 1 & P\left(x,y\right)\geq T\\ 0 & P\left(x,y\right)<T \end{array}\;$$

**Figure 3:**
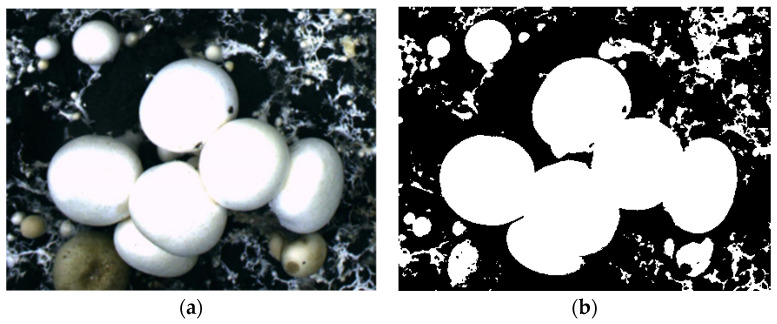
The segmented image after calculating the global gradient threshold. (**a**) Original image; (**b**) binary image after segmentation.

The binary image obtained by this method is shown in Figure 3.

The median filter is used to remove impulsive noise from the binary image of *Agaricus bisporus*, while retaining the image edge details. The median filter output is:

(6) s(x,y)=med{d(x−k,y−l),(k,l∈W)}

where d(x,y), s(x,y) are the original image and the processed image, respectively, and W is a two-dimensional template.

**Figure 4:**
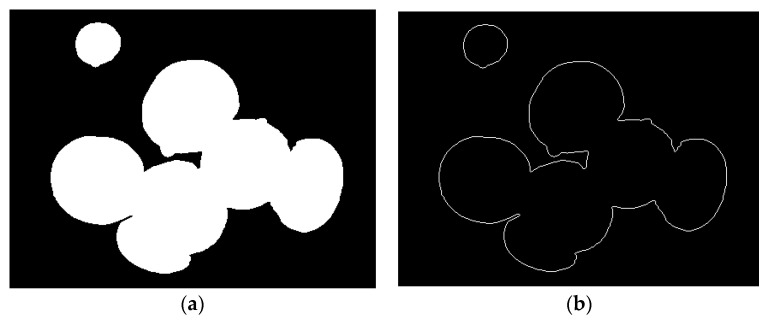
*Agaricus bisporus* area and outline. (**a**) Image after open operation; (**b**) edge detection.

Fill the holes with an area of less than 40 pixels in the *Agaricus bisporus* area, and then perform morphological processing, using a 20-pixel diameter circle and the median filtered area to open the operation to remove impurities, such as mycelium in the soil. The results are shown in Figure 4a.

Finally, the Canny operator is used to detect the edge of the processed binary image, the number of pixels in each connected area is counted, and the independent border contour blackening process with a pixel area of less than 1200 is obtained to obtain the actual contour of *Agaricus bisporus*. The result is shown in Figure 4b as follows.

### 2.2. Extraction of Convex and Concave Areas

**Figure 5:**
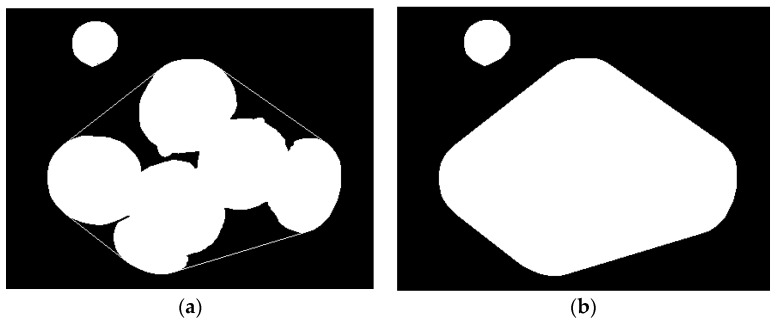
Overlapping *Agaricus bisporus* convex shell. (**a**) Convex hull curve extraction; (**b**) convex hull enclosed area.

The convex hull of the overlapping *Agaricus bisporus* area is obtained by the volume wrapped convex hull algorithm [27,28], and the outermost points of the point set in the connected area are connected to form a convex hull, as shown in Figure 5a. The convex hull curve completely encloses the *Agaricus bisporus* area and obtains the area enclosed by the convex hull curve. The result is shown in Figure 5b.

**Figure 6:**
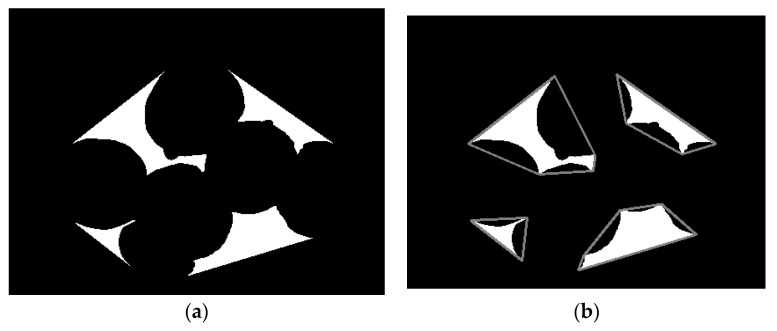
Extraction and simplification of concave areas. (**a**) Concave area; (**b**) polygon simplification.

The pixel area of the area enclosed by the convex hull curve and the overlapping spore mushroom area can be obtained as a concave area:

(7) $$\{\begin{array}{cc} h\left(x,y\right)=255 & if\left(p\left(x,y\right)=q\left(x,y\right)\right)\\ h\left(x,y\right)=0 & if\left(p\left(x,y\right)\neq q\left(x,y\right)\right) \end{array}$$

where p(x,y),q(x,y),h(x,y), respectively, represent the overlapping *Agaricus bisporus* area, convex hull area and the requested concave area. The results are shown in Figure 6a.

Convex polygon simplification for each acquired concave region and the result of polygon simplification are shown in Figure 6b.

### 2.3. Corner Detection and Contour Segmentation

Harris corner detection is used to extract the vertices of the simplified polygons in the concave area. The basic idea of Harris corner detection is to move in all directions on the image through a sliding window and compare the gray changes in pixels before and after moving. If there is a large gray change, there must be corners in the window [29].

**Figure 7:**
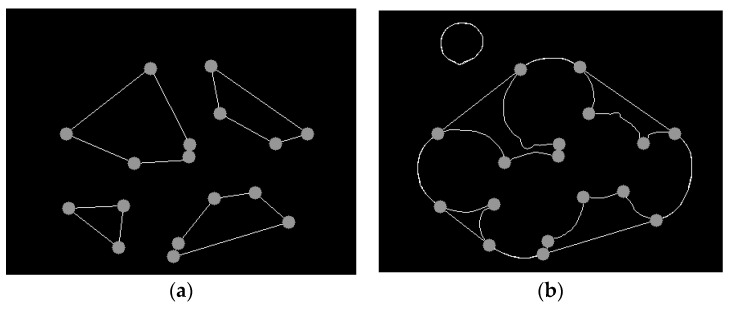
Vertex detection. (**a**) Corner detection; (**b**) corner display on the outline.

The extracted vertices are shown in Figure 7a, and the extracted vertices are displayed on the outline, as shown in Figure 7b. The eigenvalue analysis of the autocorrelation matrix M produces two eigenvalues $\left(\lambda_{1}\lambda_{2}\right)$ and two feature direction vectors. The response function R used by Harris is:

(8) $$R=\lambda_{1}\lambda_{2}-k{\left(\lambda_{1}+\lambda_{2}\right)}^{2}\;$$

**Figure 8:**
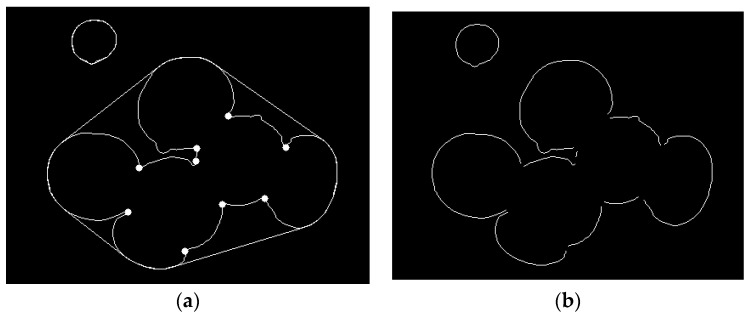
Contour segmentation of *Agaricus bisporus*. (**a**) Split point; (**b**) split contour segment.

In order to obtain the segmentation points of the overlapping *Agaricus bisporus* contours, by analyzing the distance relationship between each vertex and the convex shell contour of *Agaricus bisporus*, the vertices with a contour distance greater than 2 pixels are screened to determine the segmentation points. The segmentation point is shown in Figure 8a, and finally, the segmentation of the overlapping *Agaricus bisporus* outline is completed, as shown in Figure 8b.

## 3. Overlapping *Agaricus bisporus* Outline Grouping

After the outline of the overlapping *Agaricus bisporus* is divided, the single *Agaricus bisporus* may produce multiple contour fragments, so it is necessary to merge all the contours of the same *Agaricus bisporus*. The contour merging and grouping are abstracted into a combinatorial optimization problem, which is solved using a branch-defining algorithm [30,31,32] to obtain the optimal solution for the overlapping grouping of *Agaricus bisporus*.

### 3.1. Problem Description

Let $X=\left\{X_{1},X_{2},\cdots ,X_{D}\right\}$ be the set of D segmented segments after segmentation, and group the contours in this set into d subsets (d≤D), so that the contours belonging to the same *Agaricus bisporus* merge. Let $\varphi_{i}$ be the index number of the group to which the contour segment $X_{i}$ belongs, then use $\phi =\left\{\varphi_{1},\varphi_{2},\cdots ,\varphi_{D}\right\}$ to represent the set of all group index numbers.

The set of index numbers of all possible groupings in the contour segment is included in the real number set ℝ. The grouping criterion is given by the evaluation function J of ϕ. The evaluation function J will measure the similarity between the group ϕ and the real *Agaricus bisporus* contour segment group, so this grouping problem is to find the optimal solution set $\phi^{*}$, so that the evaluation value of the grouping criterion is the smallest:

(9) $$\phi^{*}=\underset{\phi }{\mathrm{argmin}}\;J\left(\phi ,X\right)$$

### 3.2. Branch Definition Algorithm Grouping

CONTOUR grouping is a combinatorial optimization problem. There can be at most $\overset{D}{\underset{i=1}{\sum }}\frac{i^{D}}{i!}$ solutions, and the optimal solution is determined by evaluation criterion J. However, such optimization problems will become very difficult because the number of solutions will increase exponentially [33]. The branch definition algorithm can avoid exhaustive search using the optimal solution set and can define the boundary for the evaluation function.

The partial solution calculated by the branch definition algorithm is $\phi_{g}=\left\{\varphi_{1},\varphi_{2},\cdots ,\varphi_{g}\right\},1<g<D$. Assuming that the lower bound of the value of the objective evaluation function is $b_{1},b_{2},\cdots ,b_{D}$, then:

(10) $$b_{g}=\phi_{g}\left\{\varphi_{1},\varphi_{2},\cdots ,\varphi_{g}\right\}\leq J\left(\phi ,X\right)$$

Suppose $\phi_{i}$ and $\phi_{j}$ are two sets of solutions. If the lower bound of $\phi_{i}$ is greater than the lower bound of $\phi_{j}$, and the new lower bound is $b_{i}$, then:

(11) $$b_{1}\leq b_{2}\leq \ldots \;\leq b_{D}=J\left(\phi ,X\right)$$

Assuming that B is the upper bound of the optimal solution of evaluation function J, then:

(12) $$J\left(\phi^{*},X\right)\leq B\;$$

**Figure 9:**
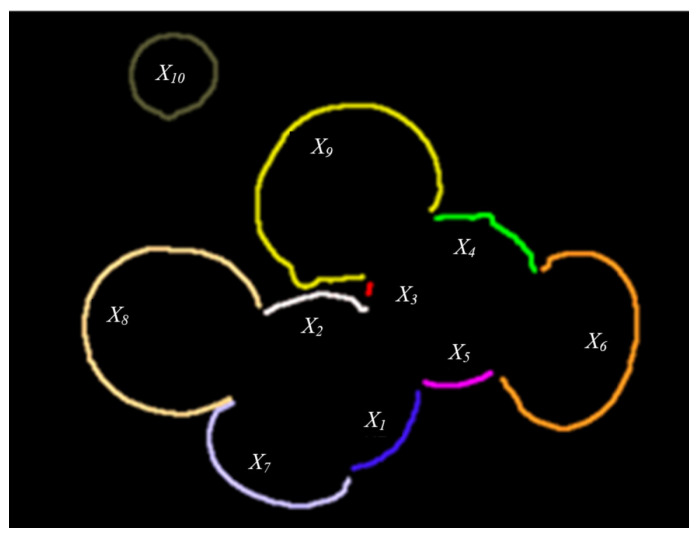
The contour representation after segmentation.

Therefore, given the restricted ranges in Equations (10) and (12), the suboptimal solution set can be removed. As shown in Figure 9, the contour segment is represented as $X=\left\{X_{1},X_{2},\cdots ,X_{10}\right\}$.

**Figure 10:**
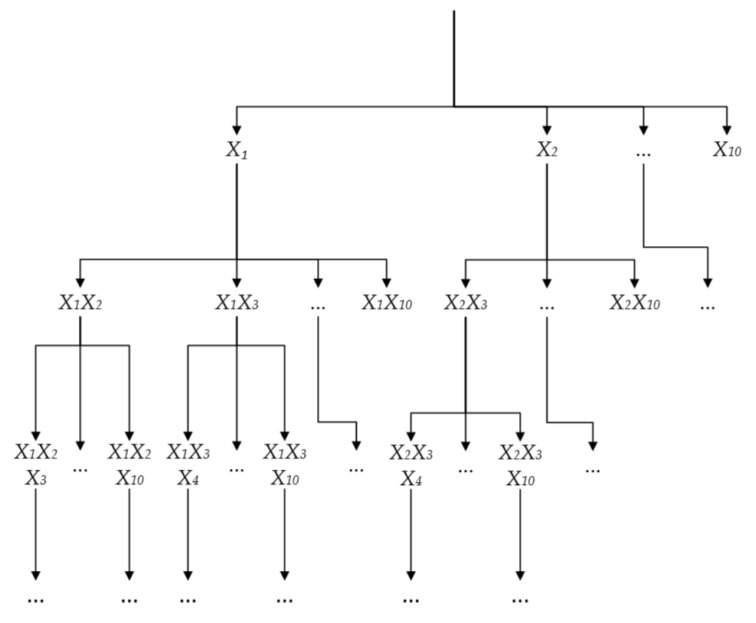
Contour fragment search tree.

The branch definition algorithm process is represented by the search tree, as shown in Figure 10. The root of the search tree represents the starting state, i.e., the 10 contour segments are all a separate group. The roots are then combined to obtain other groups by X, and each node at level d of the search tree represents a different grouping of d groups.

### 3.3. Grouping Criteria

The selection of grouping criteria greatly affects the effect of branch definition algorithm. Define the evaluation function J as:

(13) $$J=\delta J_{c}+\varepsilon J_{s}$$

where $J_{c}$, $J_{s}$ are, respectively, roundness and symmetry, δ, ε are the weight coefficients of the two parts.

Roundness represents the degree of conformity between the circle fitted by the contour and the actual contour. The contour segment $X_{i}$ consists of *n* points, and the coordinates of each point are $\left(x_{m},y_{m}\right)$ (*m* = 1, 2, …, *n*). Their corresponding points of the fitted circle are $\left(x_{j,m},y_{j,m}\right)$ (*m* = 1, 2, …, *n*), then the roundness can be expressed as:

(14) $$J_{c}=\frac{1}{n}\overset{n}{\underset{m=1}{\sum }}\sqrt{{\left(x_{m}-x_{j,m}\right)}^{2}+{\left(y_{m}-y_{j,m}\right)}^{2}}\;$$

By collecting the normal vectors of the contour segments, the symmetry centers of the contour segments $X_{i}$ and $X_{j}$ can be obtained as $P_{i}$ and $P_{j}$. As with the fast radial symmetric transformation [34], the gradient vector is replaced with the normal vector of the contour segment. The degree of symmetry $J_{s}$ can be regarded as the value of the distance between $P_{i}$ and $P_{j}$ normalized by the maximum size σ:

(15) $$J_{s}=\frac{\left|P_{i}P_{j}\right|}{\sigma }$$

**Figure 11:**
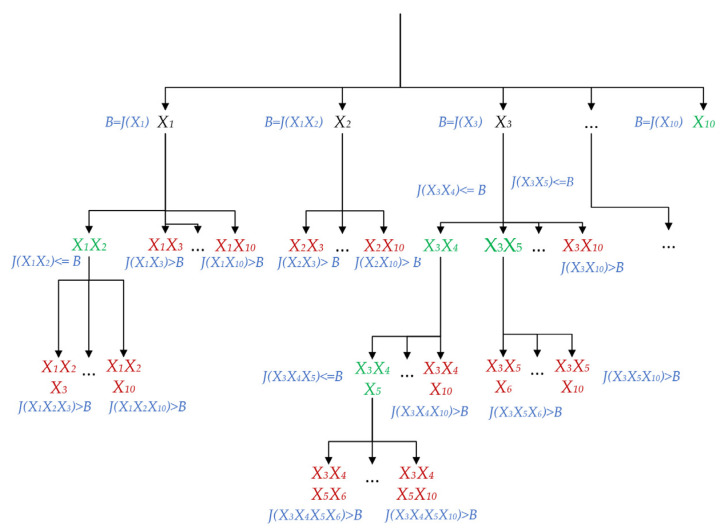
Search tree grouping expansion process.

The expansion of the search tree is controlled by the grouping criteria, and the best grouping is the node with the lowest evaluation value. As shown in Figure 11, the upper bound of the optimal group is formed after the first node is generated. If the node enters the best group, the upper bound is determined by the evaluation value of the best group. On the contrary, the upper bound is determined by the evaluation value of the node itself.

**Figure 12:**
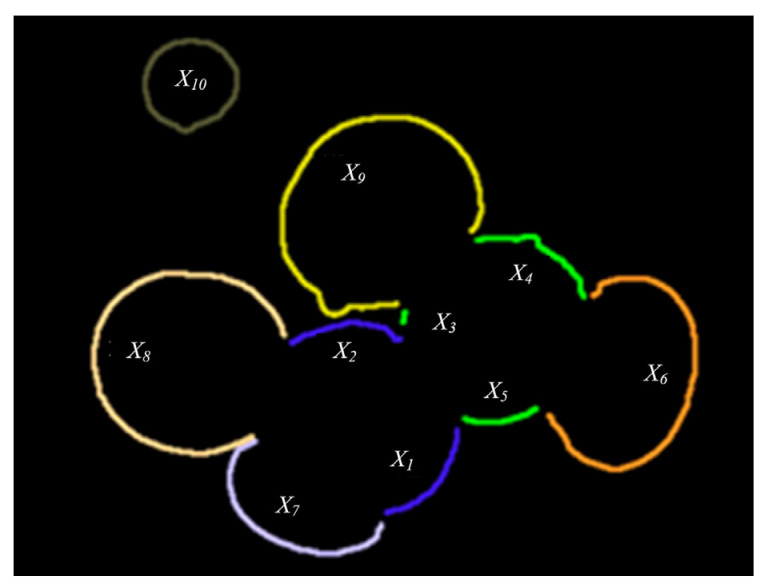
Grouped outline fragments.

The initial upper bounds of $X_{1},\;X_{3},X_{4},X_{6},X_{7},X_{8},X_{9},X_{10}$ are set to their section review value, because these nodes do not enter the best grouping. The initial definition of $X_{2}$ is $J\left(X_{1}X_{2}\right)$, because $X_{2}$ is already part of the optimal solution $\left\{X_{1}X_{2}\right\}$. When the expanded node J(ϕ,X)>B, it will stop expanding. When the value of the section reviews is less than the upper bound, the new upper bound B will be replaced. When the weighting coefficients δ and ε of roundness and symmetry are both set to 0.5, the outline grouping of the overlapping *Agaricus bisporus* is shown in Figure 12.

## 4. Reconstruction and Recognition of Overlapping *Agaricus bisporus*

**Figure 13:**
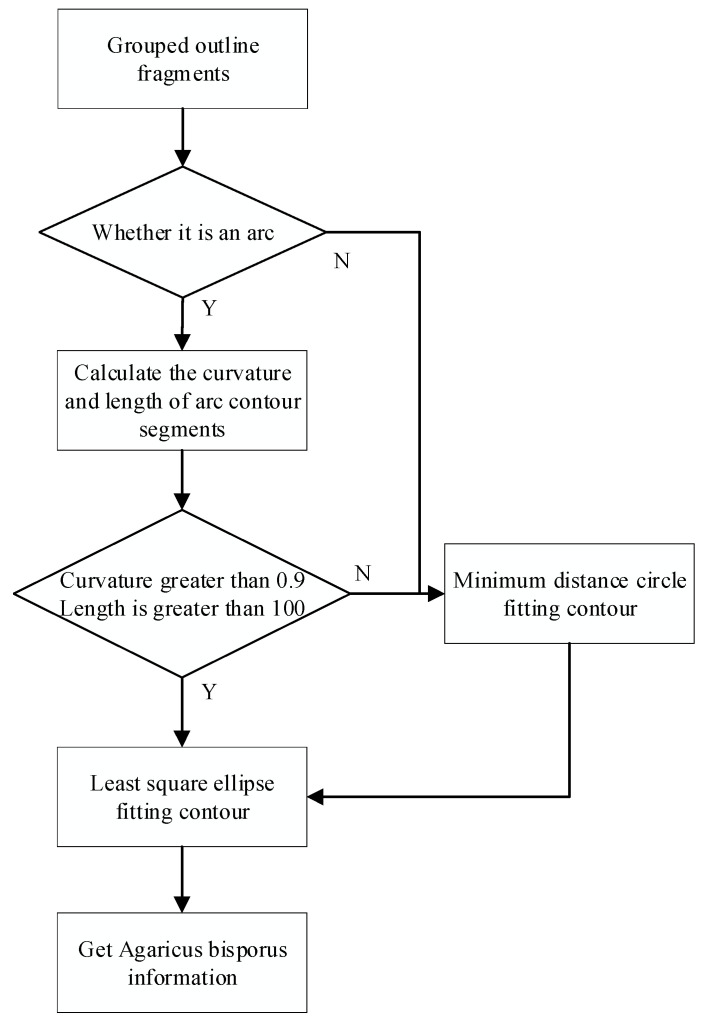
Flow chart of reconstruction contour.

Since the grouped *Agaricus bisporus* contour fragments are still not closed, in order to truly restore the *Agaricus bisporus* target, least squares ellipse fitting and minimum distance circle fitting are used to reconstruct the *Agaricus bisporus* target contour. The specific process is shown in Figure 13.

### 4.1. Least Square Ellipse Fitting Reconstruction Contour

Since the outline fragments of *Agaricus bisporus* after grouping are still scattered, if the outline is determined to be an arc, the arc height at any point on the search curve is traversed, and the height H is calculated according to the Helen formula:

(16) $$S=\sqrt{p\left(p-z\right)\left(p-v\right)\left(p-n\right)}$$

(17) $$H=\frac{S*2}{z}\;$$

where z,v,n are the three sides of the triangle formed by the points at the two ends of the contour and any point on the curve, p is the half circumference, and S is the area of the triangle.

According to the calculated bow height, the chord length of the arc is calculated, so the bow curvature Curvity can be obtained:

(18) $$R=0.5*\left(\frac{IArc^{2}}{hArc}+hArc\right)\;$$

(19) $$Curvity=\frac{1}{R}\;\;$$

where R is the bow radius of curvature, IArc is the chord length, and hArc is the bow height.

Calculate the curvature and length of each contour, and then select the contour with a curvature greater than 0.9 and a contour length greater than 200 pixels, and take N measurement points for each contour as $P_{i}\left(x_{i},y_{i}\right)\;\left(i=1,2,\ldots ,N\right)$. According to the principle of least squares, the fitting objective function is:

(20) $$F\left(A,B,C,D,E\right)=\overset{N}{\underset{i=1}{\sum }}{\left(x_{i}^{2}+Ax_{i}y_{i}+By_{i}^{2}+Cx_{i}+Dy_{i}+E\right)}^{2}\;\;$$

To minimize F, you need to:

(21) $$\frac{\partial F}{\partial A}=\frac{\partial F}{\partial B}=\frac{\partial F}{\partial C}=\frac{\partial F}{\partial D}=\frac{\partial F}{\partial E}=0\;\;$$

**Figure 14:**
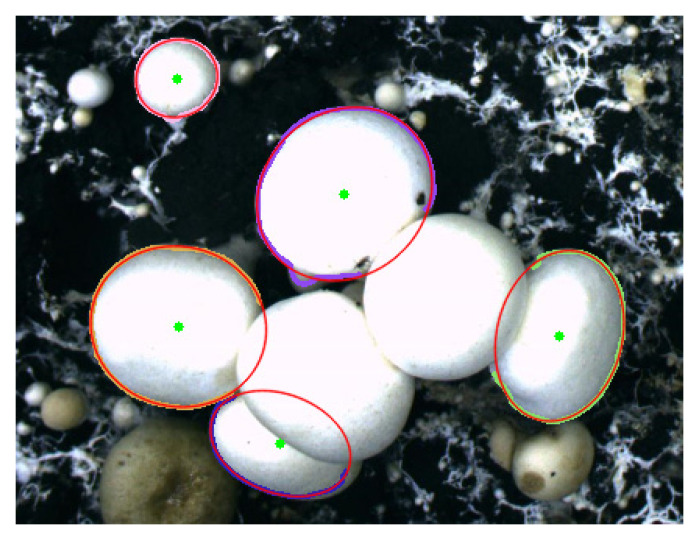
Oval profile of *Agaricus bisporus*.

The values of A,B,C,D and E can be solved. According to the characteristics of the ellipse, the ellipse position parameters $\left(\theta ,x_{0},y_{0}\right)$ and shape parameters (a,b) can be calculated to reconstruct the elliptical profile of *Agaricus bisporus*. The results are shown in Figure 14.

### 4.2. Minimum Distance Circle Fitting Contour Reconstruction

For some arc segments with low curvature or short length, if least square ellipse fitting is adopted, the center point deviation may be large, and the fitting contour deviates greatly from the actual. For the grouped and merged contours, the short-segment contours are more discrete, so for the contours of the above two cases, the circle fitting method is used to reconstruct the contours.

Least squares circle fitting is widely used, and it is very effective for data points whose errors conform to the normal distribution, but in practical applications, some interference points are often encountered. These interference points tend to deflect in a certain direction, which causes the fitted circle to deviate more. Therefore, next, the minimum distance circle fitting is used to reconstruct the *Agaricus bisporus* target.

Select n points $\left(x_{j},y_{j}\right)$ on the contour, and determine the parameters of the circle according to the sum of the absolute values of the distances from the data points to the circle, which is the following formula:

(22) $$f=\sum^{\;}\left|\sqrt{{\left(x_{j}-x_{c}\right)}^{2}+{\left(y_{j}-y_{c}\right)}^{2}}-r\right|\;$$

**Figure 15:**
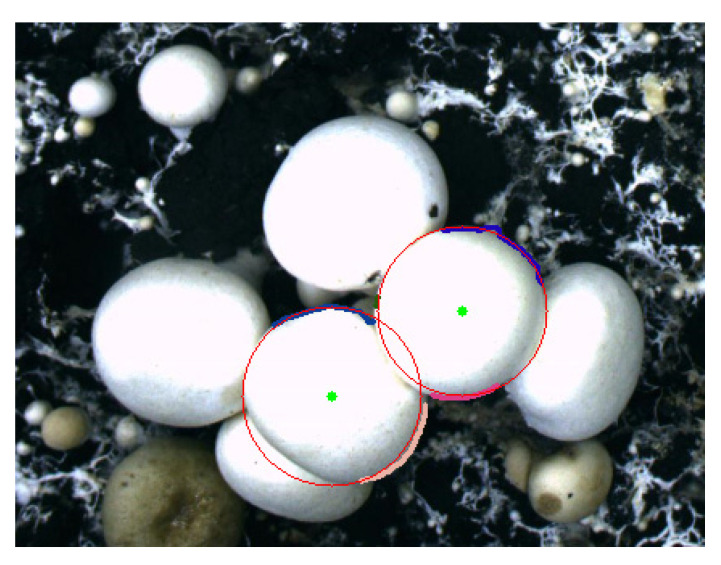
Circle outline of *Agaricus bisporus*.

The $x_{c},y_{c}$ and r that make f achieve the minimum value are the best fitting parameters. The fitting result is shown in Figure 15.

**Figure 16:**
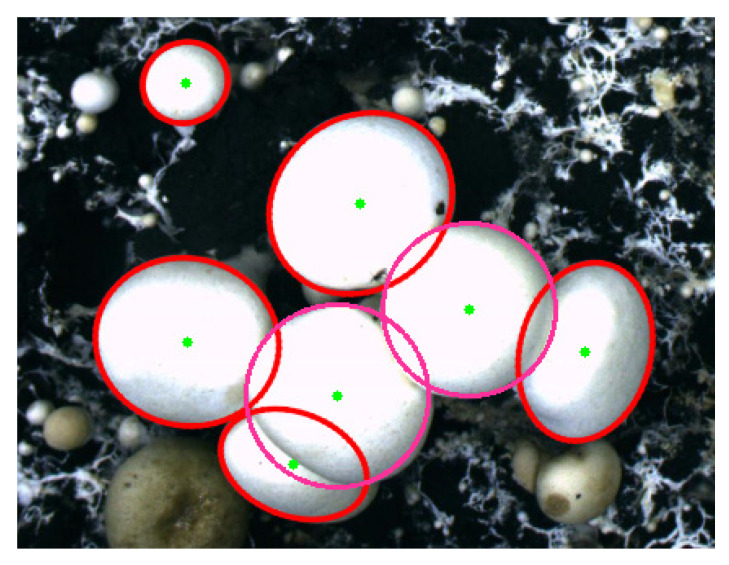
Recognition effect of *Agaricus bisporus*.

Calculate the center coordinates, long axis size and short axis size of the elliptical outline of the constructed *Agaricus bisporus*. Calculate the center coordinates and radius of the circular outline of the constructed *Agaricus bisporus*. The final recognition result of *Agaricus bisporus* is shown in Figure 16.

## 5. Experiment and Result Analysis

The experimental hardware system mainly includes a camera, lens, light source, industrial computer and so on. Among them, the industrial computer adopts Advantech embedded ARK-3500P, the operating system is Windows 7, and we use OpenCV3.4.8 library to process images in real time to obtain the picking information of *Agaricus bisporus*.

**Figure 17:**
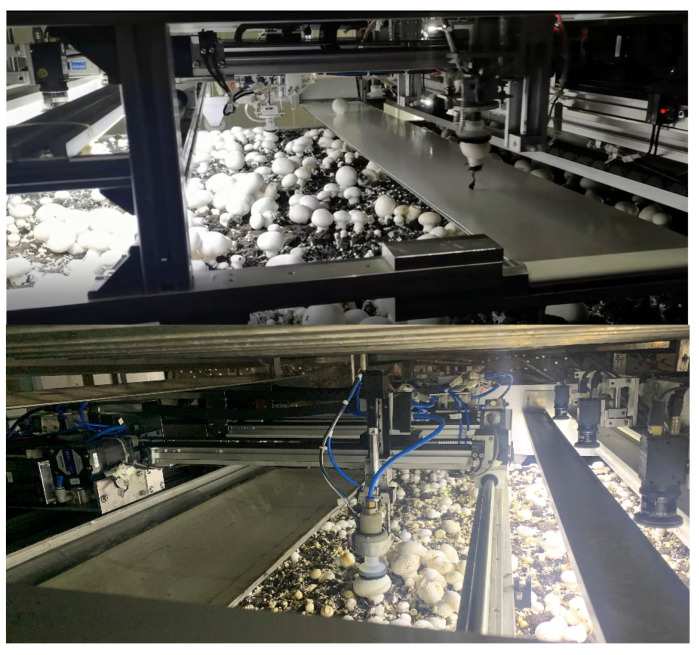
The *Agaricus bisporus* multi-arm intelligent picking robot working in the multistory shelf trays in the factory environment.

In order to verify the effectiveness of the method in this paper, sample images of *Agaricus bisporus* are all captured from the actual factory environment by the *Agaricus bisporus* multi-arm intelligent picking robot, developed by our team, as shown in Figure 17. Overlapping segmentation identification experiments of *Agaricus bisporus* were then conducted.

**Figure 18:**
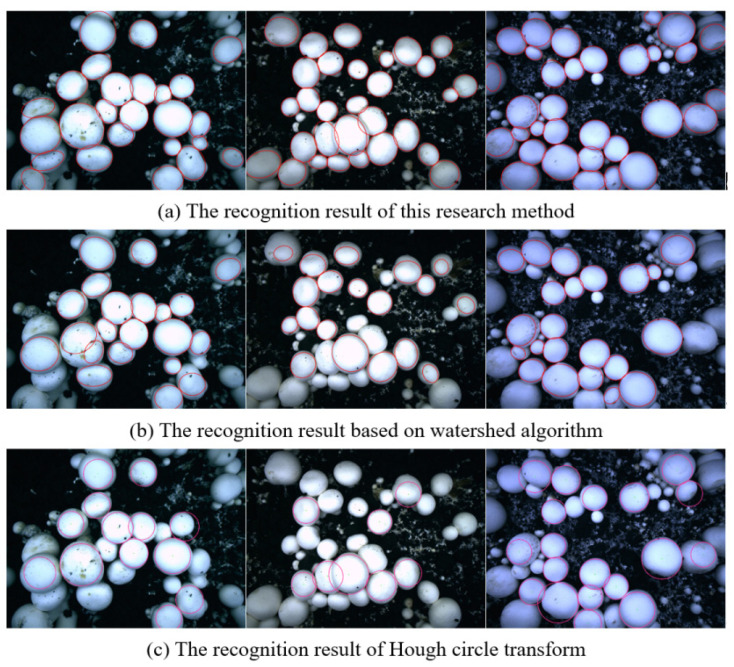
Comparison of the recognition effects of overlapping *Agaricus bisporus*.

We used the research method in this paper, the watershed algorithm based on distance transform, and the Hough circle transform algorithm to segment and identify overlapping *Agaricus bisporus*, with the comparison results shown in Figure 18. Using the research method in this paper can not only improve the adaptability to complex planting environments and overcome the influence of uneven illumination, but also accurately segment *Agaricus bisporus*, accurately reconstruct *Agaricus bisporus* contours, and meet the automatic identification and picking needs of the spore mushroom picking robot.

In the experiment, 200 sets of overlapping *Agaricus bisporus* image samples were selected for segmentation and identification and the actual number of *Agaricus bisporus* was 6109.

The research methods in this paper, the watershed algorithm based on distance transformation and the Hough circle transformation algorithm to achieve segmentation recognition, corresponding to the number of effective recognition and recognition rate comparison results, are shown in Table 1. The average recognition rate of the research method in this paper is 98.81%, which is obviously higher than the watershed algorithm and Hough circle transform algorithm based on distance transformation. It shows that the research method in this paper is effective for segmentation recognition of overlapping *Agaricus bisporus*.

In order to better evaluate the accuracy of this research method in segmentation and reconstruction of overlapping *Agaricus bisporus*, two indexes, namely, two-dimensional coordinate deviation rate *E* and recognition success rate *R*, are defined to evaluate the recognition accuracy of overlapping *Agaricus bisporus* under this research method, watershed algorithm based on distance transformation and Hough circular transformation method.

(23) $$E=\left(\left|\frac{c_{j}-c_{i}}{w}\right|+\left|\frac{r_{j}-r_{i}}{h}\right|\right)\times 100\%\;\;$$

where, $r_{j},\;c_{j}$ are the row and column coordinates of the center point of *Agaricus bisporus*, measured manually, $r_{i},\;c_{i}$ are the row and column coordinates of the central point of *Agaricus bisporus* obtained by the research method in this paper, and *W* and *H* are the width and height of the image of *Agaricus bisporus*, respectively.

If the two-dimensional coordinate deviation rate is less than 3%, it is judged that this *Agaricus bisporus* is a successful recognition, so the recognition success rate r of overlapping *Agaricus bisporus* can be calculated by Equation (24).

(24) $$R=\frac{N_{3}}{N}\times 100\%\;\;$$

where *N_3_* is the number of *Agaricus bisporus* whose deviation rate of center point coordinate recognized in the sample image is less than 3%, and *N* is the total number of *Agaricus bisporus* recognized.

In addition, the influence degree of overlapping *Agaricus bisporus* clustered can be measured by the overlapping rate *F*, which is expressed by Equation (25).

(25) $$F=\left(1-\frac{\sqrt{{\left(c_{2}-c_{1}\right)}^{2}+{\left(r_{2}-r_{1}\right)}^{2}}}{l_{1}+l_{2}}\right)\times 100\%\;$$

where, $c_{2},\;c_{1},\;r_{2},\;r_{1}$ are the manually measured row and column coordinates of two overlapping *Agaricus bisporus*,$\;l_{1},\;l_{2}$ are the pixel coordinates of the manually measured radius of the corresponding two *Agaricus bisporus*.

**Figure 19:**
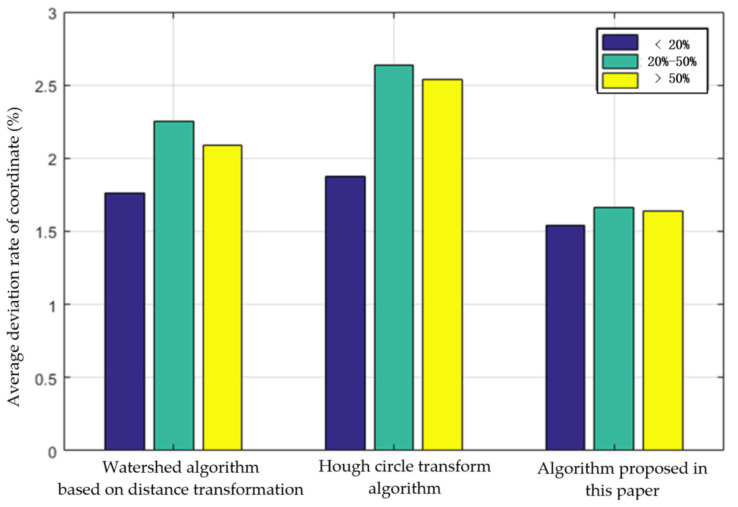
Comparison of average deviation rate of *Agaricus bisporus* coordinates under different overlap rates.

For the *Agaricus bisporus* segmented and identified by the three methods in Table 1, the average deviation rate of coordinates is counted. The statistics also count the *Agaricus bisporus* with different overlap rates. The statistical results are shown in Figure 19. Hough’s circular transformation method has the largest average coordinate deviation rate when the overlap rate of *Agaricus bisporus* is 20–50%. The average deviation rate of coordinates obtained by the algorithm in this paper is the smallest in three different intervals of *Agaricus bisporus* overlap rate.

Table 2 shows the recognition success rate (the proportion of coordinate deviation rate less than 3%) and the overall recognition success rate (the ratio of the number of successful recognitions to the total number of *Agaricus bisporus* in the original sample image) under different methods.

As shown in Table 2, the recognition success rate of the research method in this paper is 97.25%, and the overall recognition success rate is 96.09%, which is obviously higher than those of the watershed algorithm based on distance transformation and Hough circle transformation algorithm. For the statistics of *Agaricus bisporus* after segmentation and recognition, the average coordinate deviation rate of the algorithm studied in this paper is only 1.59%, and the average time is 212 ms, which is the shortest of the three methods. It shows that the research method in this paper is effective for the segmentation and recognition of overlapping *Agaricus bisporus*.

## 6. Conclusions

This paper takes *Agaricus bisporus* in a factory environment as the research object. Aiming at the complex overlapping segmentation of *Agaricus bisporus*, a segmentation identification method of overlapping *Agaricus bisporus* is proposed. According to the image edge gradient characteristics, the global gradient threshold is calculated to divide the image to obtain the binary image, and then the binary image is filtered and morphologically processed, and the contour is obtained by Canny operator edge detection. We extract the convex hull of the overlapping *Agaricus bisporus* area and the concave area to simplify the polygon. We also adopt the Harris corner to detect the vertices and determine the segmentation point, and then use the branch definition algorithm to merge and group the dispersed outlines of the same *Agaricus bisporus*. Finally, we use the least square ellipse fitting algorithm and the minimum distance circle fitting to reconstruct *Agaricus bisporus* contours. The experimental results show that the recognition rate of overlapping *Agaricus bisporus* is higher than 96%, the average coordinate deviation rate of the algorithm studied in this paper is only 1.59%, which provides picking demand information for the *Agaricus bisporus* picking robot. This method is innovative in segmentation recognition of overlapping circular fruit. However, due to the large amount of calculation in this method, it takes a long time. In future research, the algorithm needs to be improved to increase the efficiency of the algorithm.

## Figures and Tables

**Table 1:** Comparison of different methods for segmentation and identification of overlapping *Agaricus bisporus*.

Method	Number of Samples	Effective Identification Number	Recogniton Reate
Hough circle transform algorithm	6109	4338	71.01%
Watershed algorithm based on distance transformation	6109	5319	87.07%
The algorithm proposed in this article	6109	6036	98.81%

**Table 2:** Comparison of different algorithms for recognition of overlapping *Agaricus bisporus*.

Methods	Number of Segmentation and Recognition	Number of Successfully Recognited	Average Deviation Rate of Coordinates	Recognition Success Rate	Overall Recognition Success Rate	Average Time (ms)
Hough circle transform algorithm	4338	3363	2.29%	77.52%	55.05%	358
Watershed algorithm based on distance transformation	5319	4570	1.99%	85.92%	74.81%	224
The algorithm proposed in this article	6036	5870	1.59%	97.25%	96.09%	212

## Data Availability

Anyone can access our data by sending an email to szyang@sspu.edu.cn.
